# Fibroblast‐Derived TGFβ1 Regulates Skin Repair and Fibrosis

**DOI:** 10.1111/wrr.70065

**Published:** 2025-07-13

**Authors:** Sebastian Willenborg, Katrin Schönborn, Mugdha Sawant, Anna Bornikoel, Takumi Yamane, Isabel Zeinert, Beate Eckes, Sabine A. Eming, Thomas Krieg

**Affiliations:** ^1^ Department of Dermatology University of Cologne Cologne Germany; ^2^ Translational Matrix Biology University of Cologne, Medical Faculty Cologne Germany; ^3^ Department of Nutritional Science and Food Safety, Faculty of Applied Bioscience Tokyo University of Agriculture Tokyo Japan; ^4^ Cologne Excellence Cluster on Cellular Stress Responses in Ageing‐Associated Diseases (CECAD) University of Cologne Cologne Germany; ^5^ Center for Molecular Medicine (CMMC) University of Cologne Cologne Germany; ^6^ Institute of Zoology, Developmental Biology Unit University of Cologne Cologne Germany

**Keywords:** angiogenesis, fibrosis, skin repair, transforming growth factor beta

## Abstract

Activation of fibroblasts and formation of myofibroblasts are essential for granulation tissue formation following injury. In fibrotic reactions, excessive deposition of ECM by the activated fibroblasts determines scar formation and functional failure. Although these events critically depend on the activity of a plethora of growth factors and cytokines, TGFβ1 is a unique player controlling the immune response and proliferation of many cell types. Different cell types contribute to its release and activation, which is also regulated by the interaction with the ECM and by mechanical forces. The aim of this study was to elaborate whether fibroblast‐derived TGFβ1 plays a critical role during these processes. The data demonstrate a dynamic expression of TGFβ1 during tissue repair. Cell‐specific ablation of *Tgfb1* in fibroblasts revealed that deletion of TGFβ1 attenuates bleomycin‐induced skin fibrosis and perturbs maturation of granulation tissue in skin wounds. Absence of fibroblast‐derived TGFβ1 induced vascular alterations (less vascular density and branching, haemorrhage) in early wound healing. This was associated with alterations in the formation of stable ECM structure. This can be explained by paracrine regulation of endothelial cells or pericytes by fibroblast‐released TGFβ1 and by impaired expression of pro‐angiogenic factors in TGFβ1‐deficient fibroblasts. Our findings provide novel mechanistic insights into the central role of fibroblast‐derived TGFβ1 for early stages of tissue repair and fibrosis in the skin.

## 1 Introduction

Wound healing and fibrosis are similar reactions that are initiated upon tissue injury (mechanical, autoimmune, infection) with the aim to generate a functional repair. Depending on the regenerative potential of the injured tissue, repair often does not fully restore function but leads to organ malfunction and excessive scarring. Multiple diseases are associated with these aberrant healing responses and represent a huge medical and socio‐economic problem [1, 2].

The skin represents the outer barrier of the body and is challenged by a number of different stress factors that can lead to injuries. Wound healing of the skin is therefore of crucial importance. It represents a highly orchestrated event with the participation of various cell types, for example, fibroblasts, keratinocytes, endothelial cells, platelets, and a variety of immune cells including macrophages (for review see [3, 4, 5]). The repair response consists of a tightly controlled temporo‐spatial sequence of different phases starting with an initial platelet activation. This is followed by early inflammation with invasion of neutrophils and macrophages, followed by vascularization and angiogenesis. Keratinocytes are induced to migrate, ensuring a rapid wound closure, and fibroblasts, including resident and non‐resident stromal cells, are activated to produce the required extracellular matrix (ECM) constituents. Fibroblasts are also responsible for the remodelling of the newly synthesised scar tissue in later phases. Alterations in the regulated process result either in chronic, non‐healing wounds or in excessive deposition of connective tissue with an altered macromolecular organisation [6, 7]. Persistent inflammation with a resulting dense connective tissue matrix and activation of myofibroblasts are the characteristic features of excessive scarring and fibrotic responses in a variety of diseases [8]. The best possible repair response requires the interaction of all different cell types involved, the direct cell–cell contact, the crosstalk of the different cells with the ECM, and many soluble or matrix‐bound cytokines, chemokines, and growth factors.

The three TGFβ isoforms (TGFβ1, 2, 3) present in mammals are central for all cellular activities and therefore play a key role in controlling wound repair and fibrosis [9]. Consequently, several approaches have been taken to inhibit TGFβ activities aiming to treat fibrosing conditions; however, with limited success [9, 10]. In part, this can be explained by the limitations in the activities of inhibitory antibodies, by the complex actions of TGFβ and its release from multiple different cell types. In addition, TGFβ is stored in the ECM structures regulating its availability and activation, which depends on an integrin‐mediated interaction with the ECM [11, 12]. For potential specific therapeutic approaches involving TGFβ the exact role of the different cellular players and the steps involved in the activation of this potent growth factor need to be analysed in more detail.

Fibroblasts represent important target cells for TGFβ activities in tissue repair and fibrotic reactions. In response to TGFβ activity, fibroblasts are converted to myofibroblasts that are characterised by the expression and incorporation of α‐smooth muscle actin into stress fibres, thereby endowing fibroblasts with the contractile capability needed for matrix remodelling; hence αSMA is used extensively as a marker for the identification of myofibroblasts [13]. Several elegant studies have shown that the generation of the mature myofibroblast phenotype also requires mechanical tension [14, 15].

Fibroblasts interact with the surrounding ECM by specific receptors (for review see [16]), which are clustered in focal adhesions that mediate the direct contact of the cells to the ECM. These structures are composed of a complex association of many adaptor proteins, including the Integrin‐linked kinase, and allow the direct transmission of mechanical force across the cell membrane. Activation of fibroblasts in response to TGFβ is accompanied by an induced expression of ECM proteins, for example, collagen, and more recently it was shown to be associated with a reprogramming of their metabolic activities [17]. Loss of the ability to generate mechanical tension results in impaired fibroblast activation, myofibroblast formation, and reduced production of ECM proteins [18]. We have previously shown that the mechanical tension is required for TGFβ secretion by fibroblasts and that the level of Integrin linked kinase controls TGFβ secretion [19, 20]. More recently, we showed that latent TGFβ is released from fibroblasts through a secretory autophagy pathway [21], allowing a strict control of the bioavailability of TGFβ1.

The complex biology of TGFβ and the important question about the specific role of defined TGFβ1 secreting cell types as well as their target cells make it difficult to identify the exact activity of TGFβ1 in defined phases in the development of fibrosis and wound repair. In this study we address the question, which cellular players produce TGFβ1 during wound healing and fibrosis and use fibroblast‐specific inactivation of the *Tgfb1* gene to clarify that fibroblasts are not only responding to TGFβ1 signals but are significantly involved in the production of TGFβ1 during these processes. Inactivation was targeted to fibroblasts by using a Tamoxifen‐inducible Cre recombinase that is controlled by a minimal *Col1a2* promoter fused to an enhancer that restricts Cre activity to fibroblasts [22, 23]. The data presented clearly demonstrate a unique role of fibroblast‐derived TGFβ1 in mouse models of skin fibrosis and skin repair.

## 2 Materials and Methods

### 2.1 Mice

Mice with a fibroblast‐restricted ablation of *Tgfb1* (*Tgfb1* ^*fl/fl*^ *:Col1a2‐CreERT*, termed TGFβ1^FKO^) were generated by crossing *Tgfb1*‐floxed mice [24] with *Col1a2‐CreERT* mice [22]. Cre recombinase was activated by feeding tamoxifen (Envigo; 400 mg/kg) starting in week 4 until week 8 (for wounding experiments) or until the end of week 10 (bleomycin experiments). Animal housing and all experimental procedures were authorised by LANUV, North Rhine–Westphalia, Germany and University of Cologne (87–51.04.2010.A177 and 81–02.04.2018.A219).

### 2.2 Genotyping

Genomic DNA was isolated from ear punch tissue and amplified using the following PCR primer sequences: *Tgfb1*: 5′—CTT CCT AAC CCC AGA GGT GGA; 5′—CCC AGG CTA GCC TTG AAC TTC T and 5′—CAC ATT AGG TCG TGG CTA GGG; combined with 5× PCR‐Mastermix (BioBudget) using: 95°C 4 min; 95°C for 45 s, 65°C for 45 s (−0.3°C per cycle), 72°C for 1 min for 36 cycles; 72°C 5 min. PCR products of 100, 150, and 200 bp corresponding to the wildtype, the floxed, and the deleted alleles were identified by agarose gel electrophoresis. *Col1a2‐CreERT*: 5′—GGA AAT GGT TTC CCG CAG AAC CTG A; 5′—GAT GAG TTG CTT CAA AAA TCC CTT CCA, combined with 5× PCR‐Mastermix (BioBudget) using: 95°C 5 min; 95°C for 45 s, 60°C for 1 min, 72°C for 1 min for 30 cycles; 72°C 7 min. PCR product of 630 bp indicating the Cre gene was identified by agarose gel electrophoresis.

### 2.3 Bleomycin‐Induced Skin Fibrosis

6‐week‐old mice were anaesthetised with 2.25% isoflurane (NDC 66794–017‐10, Piramal) and injected intradermally with bleomycin sulphate (100 μL; 1 mg/mL in 0.9% NaCl, Medac, Wedel, Germany or Stada, Bad Vilbel, Germany) for 5 consecutive days per week for a total period of 4 weeks [18, 25]. Fibrotic lesions were excised, fixed in 4% paraformaldehyde for 2 h, and processed for paraffin embedding following standard protocols.

### 2.4 Wounding

8‐week‐old mice were anaesthetised by intraperitoneal injection of 100 mg/kg body weight Ketavet (Pfizer) and 10 mg/kg body weight Rompun 2% (Bayer). Full‐thickness punch biopsies were created on the hair‐clipped back to both sides of the spine in the scapular region using a standard biopsy puncher (pfm medical, Cologne, Germany) [26, 27]. Wounds were left uncovered to heal and were excised at 4, 7, or 14 days post injury. For histological analysis, wounds were excised at different times after injury and bisected in the caudocranial direction. Tissues were either fixed for 2 h in RotiHistofix or embedded unfixed in O.C.T. compound (Fisher Scientific).

### 2.5 Histology and Immunofluorescence

Paraffin sections were deparaffinised following standard protocols and stained with Weigert's haematoxylin (H) and eosin (E), mounted in Entellan (107,960, Sigma‐Aldrich) and imaged with a DM4000B light microscope (Leica, Wetzlar, Germany).

Dermal thickness was determined by measuring the distance between the epidermal–dermal junction and the dermal–subcutaneous fat junction in the centre of fibrotic lesions. Wound width was quantified by measuring the longest distance between left and right wound edges. Quantification was done using ImageJ software. H&E stained sections were visually inspected by five blinded experts using a scale of 1 (low) to 5 (extensive fibrosis). Immunostaining for αSMA was performed on paraffin sections using mouse αSMA Cy3‐conjugated (C6198; Sigma Aldrich 1:200; citrate buffer pH = 6 with 2100 Antigen Retriever). Integrated fluorescence intensity of the αSMA staining was measured excluding blood vessels, hair follicles, and arrector pili muscles in ImageJ software to determine αSMA positive area in the demarcated fibrotic lesion or granulation tissue. Images were captured using a fluorescence microscope (Keyence, BIOREVO BZ‐9000E). For CD31 immunofluorescence staining, cryostat sections (10 μm thick) from OCT embedded tissues were fixed in acetone and blocked in 10% normal goat serum in PBS for 1 h at RT. Slides were incubated with anti‐CD31 (BD Biosciences), diluted 1:50 in blocking buffer overnight at 4°C. Bound primary antibodies were detected by Alexa Fluor 488‐conjugated secondary antibody (Thermo Fisher Scientific), followed by counterstaining with DAPI (Invitrogen). Slides were covered with coverslips in mounting medium (glycerol gelatine). Stained sections were analysed using the Keyence BZ‐9000 fluorescence microscope and the BZ‐II viewer software. Photographs were further processed with Adobe Photoshop 7.0 (Adobe Systems) and the relative proportion of the CD31^+^ area per hpf was analysed with ImageJ. Phospho‐SMAD2 immunostaining was performed on paraffin sections (anti‐pSMAD2 antibody from Cell Signaling Technology; 1:250; citrate buffer pH = 6 as antigen retriever).

### 2.6 Picrosirius Red Staining

Picrosirius red staining of wounds and fibrotic lesions was done using a staining kit (abcam ab150681). Briefly, sections were deparaffinised and hydrated in distilled water, incubated with Picrosirius Red solution for 60 min, rinsed twice with acetic acid solution, once with absolute alcohol, and mounted with Pertex (41–4012‐00; Medite). Images were captured under polarised light using DM4000B light microscope (Leica, Wetzlar, Germany). Percentage of red and green collagen fibres was quantified using ImageJ software.

### 2.7 Masson's Trichrome Staining

Deparaffinised tissue sections were rinsed in distilled water and incubated in resorcinol‐fuchsin solution for 15 min, in iron‐haematoxylin solution for 3 min, rinsed in distilled water, briefly immersed in HCl, rinsed with distilled water, and incubated in Goldner solution I for 1 min. After rinsing in distilled water, acetic acid, and distilled water, the sections were incubated in Goldner solution II for 5 min, rinsed in distilled water, acetic acid, and distilled water, and incubated for 4 min in Goldner solution III. After rinsing again in distilled water, acetic acid, and distilled water, sections were dehydrated, cleared, and mounted in Entellan. Masson's trichrome staining was used to visualise erythrocytes in granulation tissue. The area in granulation tissue covered by erythrocytes was determined using Fiji software.

### 2.8 Isolation of Primary Dermal Fibroblasts From Adult Mice

Back skin including the epidermis of Tamoxifen‐fed mice, sacrificed at the end of 10 weeks of age, was incubated in sterile DMEM with 0.4 μg/mL CaCl_2_ and in 0.25% trypsin (5 mL buffer/mouse) containing 4 mg/mL collagenase 4 (Worthington) for 2 h at 37°C with vortexing. Liberated cells were passed through a 70 μm cell strainer and collected (1000 rpm, 5 min) and cultured in complete DMEM with 10% FCS. For further analysis, cells were cultured for 72 h in serum replacement medium containing 46.5% AIM‐V (12055–091, Gibco); 46.5% DMEM, 5% RPMI (61870–010, Gibco), supplemented with 100 U/mL penicillin, 100 μg/mL streptomycin, 2 mM L‐glutamine, 50 μg/mL Na‐ascorbate, and 1% non‐essential amino acids (N7145, Sigma‐Aldrich).

### 2.9 Flow Cytometry

Single‐cell suspensions of wound tissue and normal skin were prepared by a combination of enzymatic digestion (Liberase Blendzyme, Roche Applied Science) and mechanical disruption (Medimachine System, BD Biosciences). Excised tissue (without removing the epidermis) was minced with a scalpel, placed in 1.2 mL DMEM medium supplemented with 30 mg/mL Liberase TM Research Grade (Roche), and incubated at 37°C for 90 min (shaking). Digested wound tissue was mechanically disrupted (5 min using Medimachine System, BD Biosciences). Cells were passed through 70 μm and 40 μm cell strainers and washed with ice‐cold PBS/1% BSA/2 mM EDTA. Fc receptors were blocked with anti‐CD16/CD32 (1:50) and cells were stained with phycoerythrin (PE)‐conjugated anti‐CD11b (1:300) and allophycocyanin (APC)‐conjugated anti‐CD31 (1:100) in PBS/1% BSA/2 mM EDTA. Dead cells were excluded using 7‐AAD (all from Thermo Fisher Scientific). Cells were sorted using a FACSAria III system equipped with FACSDiva Version 6.1.1 software (BD Biosciences). FACS data were analysed by FlowJo Version 10.7.1 (FlowJo).

### 2.10 Quantitative Real‐Time PCR

FACS‐sorted CD11b^−^CD31^−^eYFP^+^ cells were lysed in 350 μL RLT buffer (Qiagen). Total RNA was isolated using the RNeasy Plus Micro kit (Qiagen) according to the manufacturer's instructions. Reverse transcription of isolated RNA was performed using the High Capacity cDNA RT Kit (Thermo Fisher Scientific). Amplification reactions (triplicates) were set up using the PowerSYBR Green PCR Master Mix and Quant‐StudioTM 5 Real‐time PCR system (Thermo Fisher Scientific). The comparative method of relative quantification (2^−ΔΔCt^) was used to calculate the expression level of the target gene normalised to *Gapdh* as housekeeping gene. The following primer pairs were used: *Tgfb1* forward: 5′ TGGAGCAACATGTGGAACTC, *Tgfb1* reverse: 5′ GTCAGCAGCCGGTTACCA, *Plod2* forward: 5′ TCCTGATGGGTACTATGCTCG CTCT, *Plod2* reverse: 5′ CGGAGTAGGGGAGTCTTTTTCCCTT, *Comp* forward: 5′ GCGCCAGTGTCGCAAGGACAA, *Comp* reverse: 5′ TGGGTTTCGAACCAGCGGGC, *Acta2* forward: 5′ TCGCTGTCAGGAACCCTGAGACG, *Acta*2 reverse: 5′ CACCAGCG AAGCCGGCCTTAC.

### 2.11 TGFβ1 ELISA

Primary dermal fibroblasts were isolated from homeostatic skin of adult mice and cultivated as described above (see paragraph “Isolation of primary dermal fibroblasts from adult mice”). Supernatants were collected and centrifuged to eliminate cell debris. TGFβ1 secreted by fibroblasts was quantified in biological triplicates using TGF‐beta 1 Quantikine ELISA Kit (R&D Systems, MB100B). ELISA was performed according to the manufacturer's instructions.

### 2.12 RNA‐Seq Analysis

Primary dermal fibroblasts were isolated from homeostatic skin of adult mice and cultivated as described above (see paragraph “Isolation of primary dermal fibroblasts from adult mice”). RNA was extracted from primary dermal fibroblasts using RNeasy Plus Micro Kit (Qiagen, cat. no. 74034) according to manufacturer's protocol. All samples had an RNA integrity number (RIN) ≥ 9. Paired‐end sequencing was performed at Novogene (UK) on Illumina platform (PE150, 20 million reads). HISAT2 was used for alignment to a reference genome ( *Mus musculus* , GRCm38/mm10). Statistical analysis and visualisation of the results was done with R 4.3.2 [28] using R Studio 2023.09.1 + 494 (https://www.r‐project.org/) and the R packages: ggplot2 3.4.4 [29], ggh4x 0.2.7 [30], ggrepel 0.9.4 [31], circlize 0.4.15 [32], stringr 1.5.1 [33], dyplr 1.1.4 [34], DESeq2 1.42.0 [35], org.Mm.eg.db 3.18.0 [36], clusterProfiler 4.10.0 [37], svglite 2.1.3 [38]. To reduce noise by lowly expressed transcripts, we filtered out transcripts whose overall sum of counts across all samples was below the threshold of 5× the number of samples. Normalised counts were calculated according to the DESeq2 standard analysis workflow and obtained from the multiple‐testing‐corrected (Benjamini‐Hochberg) DESeq2 results using an alpha‐level of 0.05 (adjusted to multiple comparisons) and log2 fold change threshold of 0.6. Gene set enrichment analysis for gene ontology (GO) terms was performed with clusterProfiler using default settings. RNA‐Seq data can be accessed under NCBI GEO: GSE298182.

### 2.13 Statistical Analyses

Appropriate tests as specified in figure legends to determine the statistical significance of the data were applied using GraphPad Prism (GraphPad, v.7). Parametric tests were used for data showing normal distribution (D'Agostino–Pearson test) and non‐parametric tests for otherwise.

## 3 Results

### 3.1 Fibroblast‐Specific TGFβ1 Deletion Attenuates Bleomycin‐Induced Dermal Fibrosis in Mice

**FIGURE 1:**
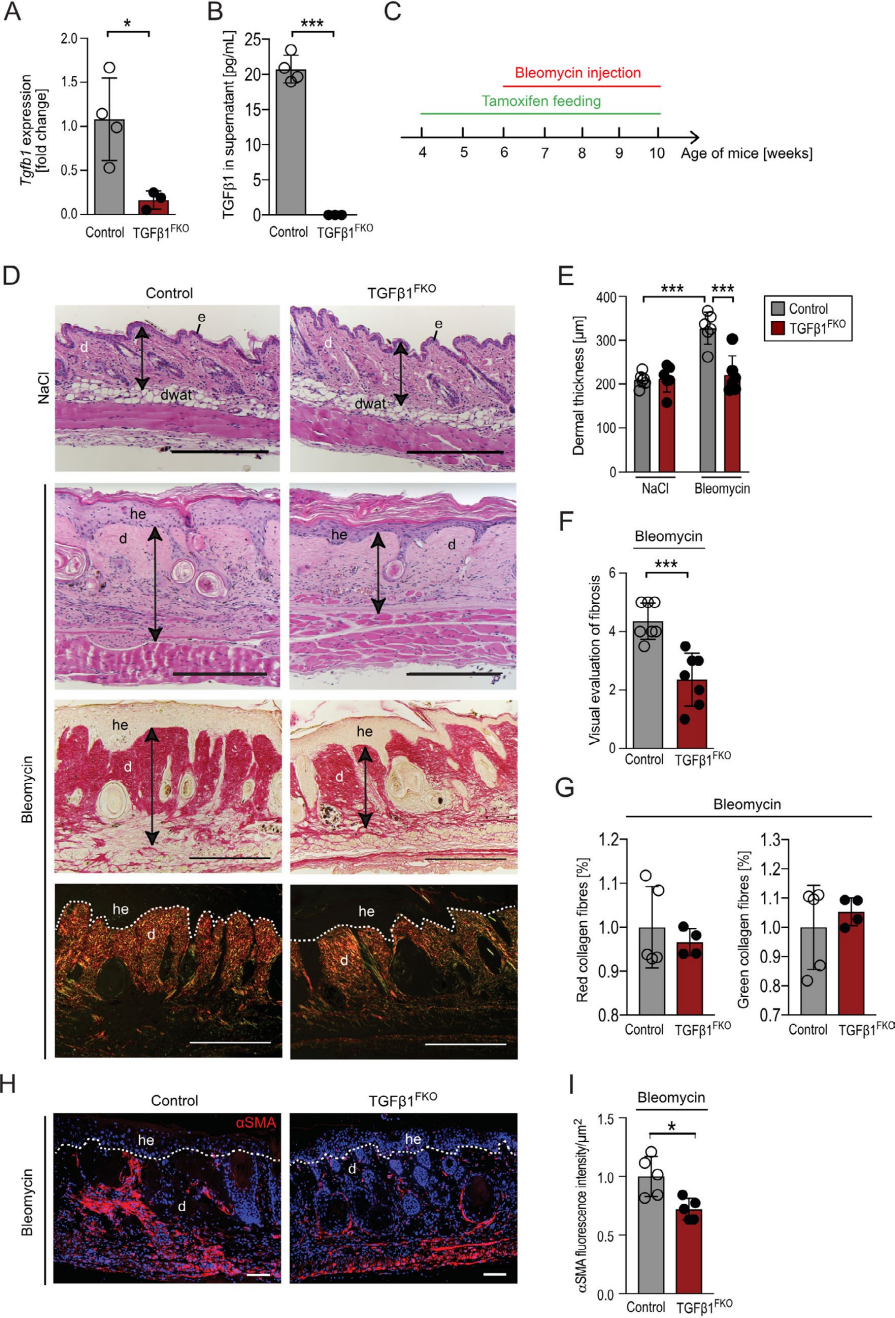
Fibroblast‐specific TGFβ1 plays a crucial role in bleomycin‐induced dermal fibrosis in mice. (A) *Tgfb1* gene expression in dermal fibroblasts isolated from tamoxifen‐fed TGFβ1^FKO^ and control mice. (B) TGFβ1 protein concentration in supernatants of dermal fibroblasts isolated from tamoxifen‐fed TGFβ1^FKO^ and control mice. (C) Timeline of tamoxifen feeding and bleomycin injections in TGFβ1^FKO^ and control mice. (D) (Upper panels) Representative images of H&E‐stained skin sections of tamoxifen‐treated TGFβ1^FKO^ and control mice subjected to intradermal NaCl or bleomycin injection. (Lower panels) Representative images of picrosirius‐red‐stained fibrotic lesions in tamoxifen‐treated TGFβ1^FKO^ and control mice subjected to intradermal bleomycin injections. Images were taken by bright field microscopy or in polarised light. Scale bar: 300 μm. (E) Quantification of dermal thickness (*n* = 6 biological replicates) and (F) visual evaluation of fibrosis in tamoxifen‐treated TGFβ1^FKO^ and control mice subjected to intradermal bleomycin or NaCl injection. (G) Analysis of the proportion of red and green collagen fibres in picrosirius red‐stained wound sections in polarised light in fibrotic lesions of tamoxifen‐treated TGFβ1^FKO^ and control mice. Data are normalised to the control group. *n* = 4–5 biological replicates. (H) Representative αSMA immunofluorescence images in fibrotic lesions of tamoxifen‐treated TGFβ1^FKO^ and control mice. DAPI was used to stain nuclei. Scale bar: 50 μm. (I) Quantification of αSMA immunofluorescence intensity in fibrotic lesions of tamoxifen‐treated TGFβ1^FKO^ and control mice. Quantification was restricted to dermal fibroblasts, thus excluding hair follicles, arrector pili muscle and blood vessels. (D, H) d, dermis; dwat, dermal white adipose tissue; e, epidermis; he, hyperproliferative epithelium. Data are shown as mean ± SD. *p < 0.05, **p < 0.01, ***p < 0.001 by Mann–Whitney *U* test (A, B, F, G, I) or by One‐way ANOVA with Tukey's Multiple Comparison Test (E).

To analyse the functional impact of fibroblast‐derived TGFβ1 on skin repair and fibrosis, we generated mice with tamoxifen‐inducible fibroblast‐specific *Tgfb1* deficiency (TGFβ1^FKO^ mice; *Tgfb1* ^*fl/fl*^ *:Col1a2‐CreERT*) [22, 24]. In *Tgfb1* ^*fl/fl*^ *:Col1a2‐CreERT* mice, Cre recombinase is expressed under control of the collagen type I alpha 2 promoter and can be activated by application of tamoxifen, leading to deletion of the *Tgfb1* gene. Controls expressed the floxed *Tgfb1* alleles but no Cre recombinase. As expected, *Tgfb1* ^*fl/fl*^ *:Col1a2‐CreERT* and control mice (*Tgfb1* ^*fl/fl*^) developed normally and did not develop spontaneous phenotypes. To assess *Tgfb1* gene deletion efficiency, we isolated skin fibroblasts from TGFβ1^FKO^ and control mice after 4 weeks of tamoxifen feeding. qRT‐PCR analysis of isolated skin fibroblasts and ELISA of cell culture supernatants showed highly efficient deletion of TGFβ1 transcripts and protein in fibroblasts isolated from TGFβ1^FKO^ mice compared with controls (Figure 1A,B). Next, we subjected tamoxifen‐fed TGFβ1^FKO^ and control mice to repeated intradermal (i.d.) injections of bleomycin in order to induce skin fibrosis or of sterile NaCl solution as control (Figure 1C). After 4 weeks of bleomycin‐ or NaCl treatment, haematoxylin and eosin (H&E)–stained sections of injected tissues from TGFβ1^FKO^ and control mice were analysed. Over the course of the experiment, we found no signs of skin fibrosis in NaCl‐treated controls and no obvious differences in skin morphology between tamoxifen‐fed and NaCl‐treated TGFβ1^FKO^ and control mice (Figure 1D upper panel, 1E). Instead, tissue from bleomycin‐treated control mice showed clear signs of skin fibrosis including dermal thickening, hyperproliferation of the epidermis, degeneration of hair follicles, and replacement of dermal white adipose tissue with collagen when compared with the NaCl‐treated control tissue (Figure 1D–F). By contrast, dermal thickness and fibrosis score based on visual evaluation were significantly lower in bleomycin‐treated TGFβ1^FKO^ mice than in controls (Figure 1D–F). Lower dermal thickness in fibrotic lesions of TGFβ1^FKO^ mice was confirmed in Picrosirius Red‐stained tissue sections (Figure 1D lower panels), which in polarised light revealed that the proportions of red and green collagen fibres in fibrotic lesions were similar in TGFβ1^FKO^ mice and control mice (Figure 1D lower panels, 1G).

Interestingly, bleomycin treatment resulted in an accumulation of αSMA‐positive myofibroblasts in control mice, which was significantly lower in TGFβ1^FKO^ mice (Figure 1H,I). Taken together, our findings identify fibroblast‐derived TGFβ1 as a critical driver of bleomycin‐induced skin fibrosis.

### 3.2 Fibroblasts Express Tgfb1 and Other Pro‐Fibrotic Genes During Skin Wound Healing

**FIGURE 2:**
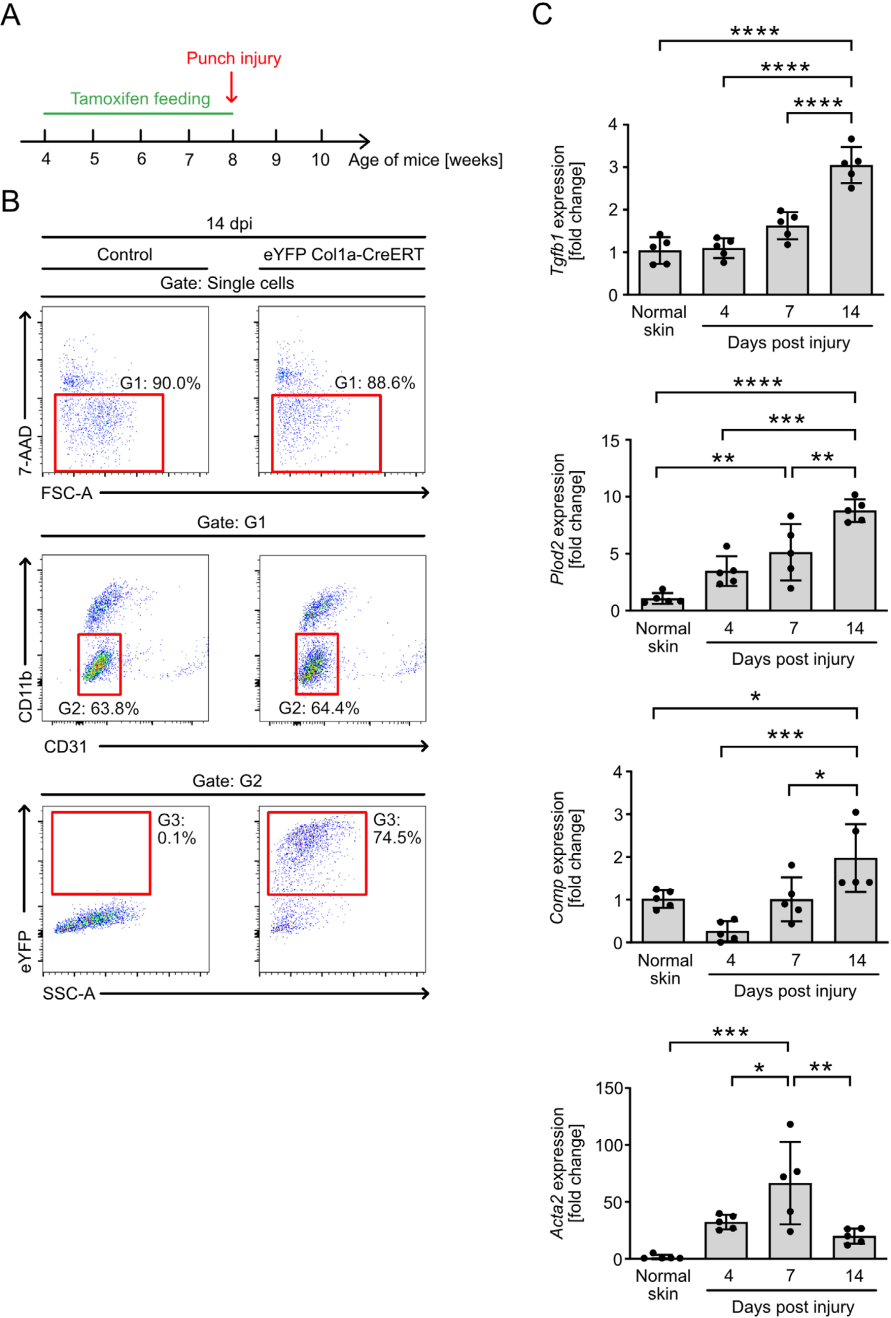
Fibroblasts express *Tgfb1* and other pro‐fibrotic genes during skin wound healing. (A) Timeline of wound healing experiments in *R26‐eyfp:Col1a2‐CreERT* reporter mice. (B) Gating strategy for flow cytometric sorting of wound fibroblasts shown as an example for day 14 post injury. Wound cells isolated from *R26‐eyfp:Col1a2‐CreERT* and Cre‐negative control mice were analysed. Single viable (7‐AAD^−^) cells were gated (Gate G1) and analysed for CD11b and CD31 expression. 7‐AAD^−^CD11b^−^CD31^−^ cells were gated (Gate G2) and analysed for eYFP fluorescence (Gate G3). 7‐AAD^−^CD11b^−^CD31^−^eYFP^+^ cells were considered as wound fibroblasts, gated and sorted. dpi, days post injury; FSC, forward scatter; SSC, sideward scatter. (C) Gene expression in sorted fibroblasts (7‐AAD^−^CD11b^−^CD31^−^eYFP^+^) isolated from normal skin and wound tissue of *R26‐eyfp:Col1a2‐CreERT* reporter mice. Data are normalised to fibroblasts isolated from normal skin. *n* = 5 biological replicates per time‐point. Data are shown as mean ± SD. *p < 0.05, **p < 0.01, ***p < 0.001, ****p < 0.0001 by One‐way ANOVA with Tukey's multiple comparison test.

To assess the expression of *Tgfb1* in fibroblasts during physiological skin repair, we made use of *R26‐eyfp:Col1a2‐CreERT* reporter mice [39]. In *R26‐eyfp:Col1a2‐CreERT* mice, Cre‐mediated excision of a STOP cassette results in the expression of eYFP. We fed *R26‐eyfp:Col1a2‐CreERT* and control (*R26‐eyfp*) mice with tamoxifen for 4 weeks and then subjected the mice to a model of full‐thickness excisional skin injury to the back skin (Figure 2A). We generated single‐cell suspensions from wound tissue during the early inflammatory phase (4 days post‐injury [dpi]), the mid tissue‐forming phase (7 dpi) and the late scar‐forming phase (14 dpi) as well as from unwounded skin, and sorted CD11b^−^CD31^−^eYFP^+^ cells (considered as fibroblasts) by flow cytometry (Figure 2B). Sorted CD11b^−^CD31^−^eYFP^+^ cells were subjected to quantitative Real‐time‐PCR (qRT‐PCR) analysis. Gene expression was normalised to CD11b^−^CD31^−^eYFP^+^ cells isolated from normal skin. Analysis revealed a gradual increase of *Tgfb1* expression and of other pro‐fibrotic genes known to be expressed in fibroblasts, such as *Plod2* (encoding lysyl hydroxylase 2) and *Comp* (encoding cartilage oligomeric matrix protein) from the inflammatory towards the scar‐forming phase (Figure 2C). Importantly, the expression of *Acta2* (encoding α‐smooth muscle actin), a hallmark gene of myofibroblasts, was induced in CD11b^−^CD31^−^eYFP^+^ cells at 4 dpi when compared with normal skin, peaked at 7 dpi, and declined towards 14 dpi. This shows that the expression of *Acta2* in CD11b^−^CD31^−^eYFP^+^ cells parallels the dynamics of myofibroblast accumulation in wound tissue over the healing response, as described previously [26, 40, 41, 42]. Taken together, our findings in *R26‐eyfp:Col1a2‐CreERT* reporter mice show that fibroblasts express *Tgfb1* dynamically during skin wound healing.

### 3.3 Loss of Fibroblast‐Derived TGFβ1 Perturbs Maturation of Granulation Tissue in Skin Wounds

**FIGURE 3:**
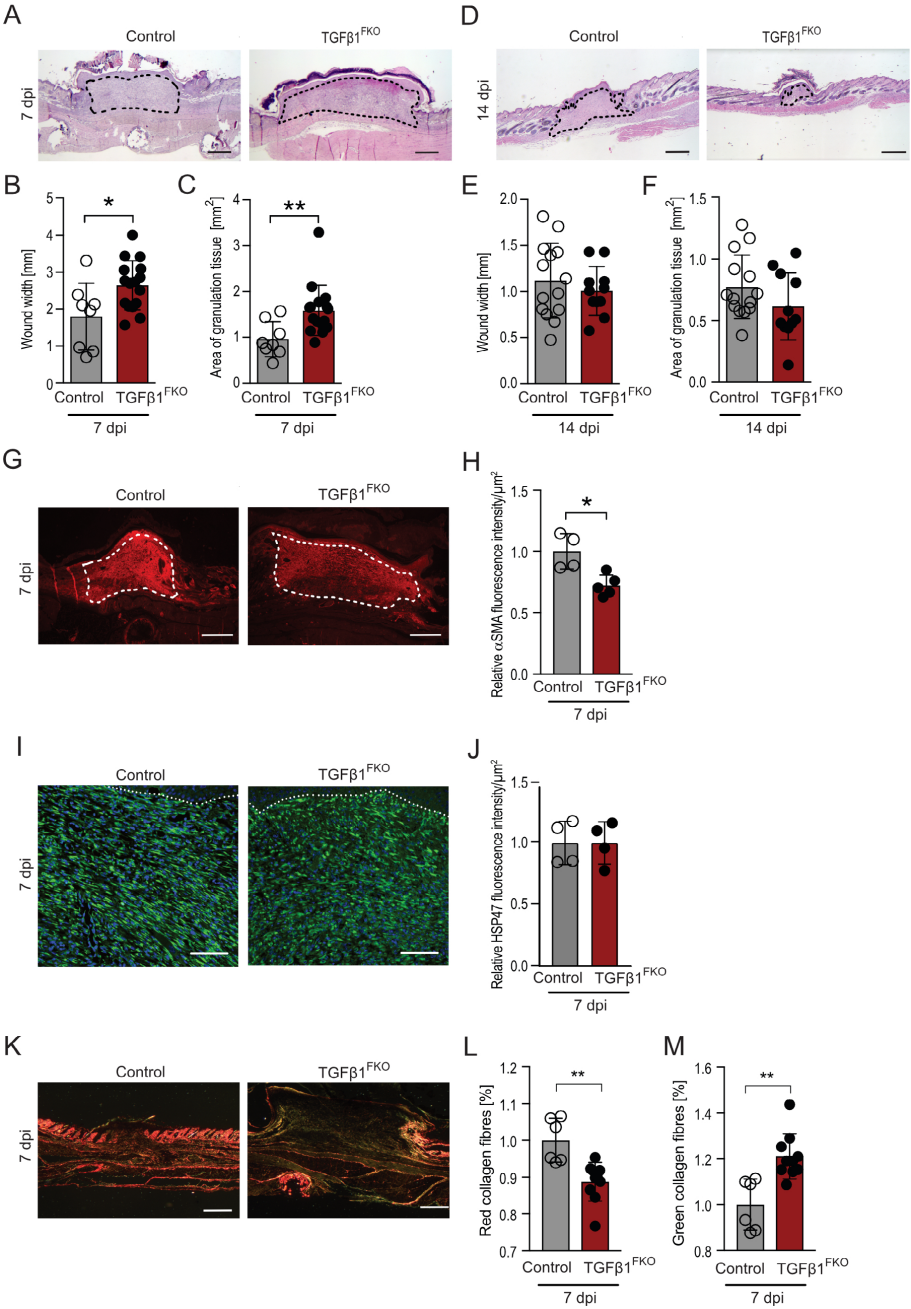
Loss of fibroblast‐derived TGFβ1 impairs skin wound healing. Representative H&E stainings of wound tissue at 7 (A) and (D) 14 dpi in tamoxifen‐treated TGFβ1^FKO^ and control mice and quantification of (B, E) wound width and (C, F) area of granulation tissue. Dashed line indicates granulation tissue. *n* = 8–11 biological replicates. (G) Representative αSMA immunofluorescence images in wound tissue at 7 dpi from tamoxifen‐treated TGFβ1^FKO^ and control mice. Dashed line indicates granulation tissue. (H) Quantification of αSMA immunofluorescence intensity in wound tissue at 7 dpi from tamoxifen‐treated TGFβ1^FKO^ and control mice. The data are normalised to the control group. *n* = 4–5 biological replicates. (I) Representative immunostainings against HSP47 of wound tissues at 7 dpi from tamoxifen‐treated TGFβ1^FKO^ and control mice. DAPI was used to stain the nuclei. Scale bar = 100 μm. (K) Representative images of picrosirius red staining in polarised light of granulation tissue at 7 dpi in tamoxifen‐treated TGFβ1^FKO^ and control mice. Analysis of the proportion of red (L) and green (M) collagen fibres in picrosirius red‐stained wound sections in polarised light at 7 dpi in tamoxifen‐treated TGFβ1^FKO^ and control mice. *n* = 6–9 biological replicates. (A, D, K) Scale bars = 500 μm. Data are shown as mean ± SD. *p < 0.05, **p < 0.01 by Mann–Whitney *U* test.

The considerable alleviation of dermal fibrosis in the absence of fibroblast‐derived TGFβ1 prompted us to ask if the formation of new dermal connective tissue after skin injury may also be affected. To address this question, we fed TGFβ1^FKO^ and control mice with tamoxifen for 4 weeks and subjected the mice to full‐thickness excisional punch injury as described above (Figure 2A). Histological analysis of H&E‐stained wound sections revealed a significantly higher wound width and area of granulation tissue at 7 dpi in TGFβ1^FKO^ mice compared with controls (Figure 3A–C). In addition, the granulation tissue at 7 dpi in TGFβ1^FKO^ mice showed signs of haemorrhage in the upper part of the wound that were not visible in control wounds (Figure 3A). At 14 dpi, wound width and area of granulation tissue in TGFβ1^FKO^ mice were similar to controls, indicating that loss of fibroblast‐specific TGFβ1 transiently altered the repair process (Figure 3D–F). The higher wound width observed in TGFβ1^FKO^ mice compared with controls suggests impaired wound contraction in TGFβ1^FKO^ mice mediated by contractile αSMA^+^ myofibroblasts. We hypothesized that fibroblast‐specific TGFβ1 deletion leads to impaired expression of αSMA, a downstream target of TGFβ1. Indeed, αSMA immunostaining of wound sections at 7 dpi revealed a significantly lower αSMA fluorescence intensity in the granulation tissue of TGFβ1^FKO^ mice compared with controls, reflecting lower αSMA expression in wounds of TGFβ1^FKO^ mice versus controls (Figure 3G,H). At 14 dpi, αSMA was barely detectable in the wounds of both control and TGFβ1^FKO^ mice, with no significant differences between the two genotypes (Figure S1A,B). We asked whether fibroblast numbers are regulated by fibroblast‐specific TGFβ1 and subjected wound sections to immunostaining against heat shock protein 47 (HSP47), a collagen‐specific chaperone and fibroblast marker. Analysis revealed similar HSP47 signal intensity in the granulation tissue of control and TGFβ1^FKO^ mice, suggesting that fibroblast‐derived TGFβ1 is dispensable for proliferation or recruitment of wound fibroblasts but critical for the differentiation of fibroblasts into myofibroblasts (Figure 3I,J).

Besides αSMA, collagen is an important downstream target of TGFβ1 signalling which controls wound closure. Collagen deposited into the ECM of the granulation tissue controls the tensile strength of the wound, which may consequently impact wound width and scar quality. To assess collagen deposition, we subjected wound sections from control and TGFβ1^FKO^ mice at 7 dpi to Picrosirius Red staining and analysed the stained sections in polarised light (Figure 3K). The percentage of red collagen fibres depicting mature collagen type I fibres was significantly lower in TGFβ1^FKO^ mice wounds compared with controls (Figure 3K,L). Of note, the percentage of green collagen fibres denoting thinner collagen fibres, possibly containing collagen type III that is synthesised in early wound healing, was significantly higher in the granulation tissue of TGFβ1^FKO^ mice wounds when compared with controls (Figure 3M). Taken together, our findings show that loss of fibroblast‐derived TGFβ1 perturbs maturation of granulation tissue.

### 3.4 Fibroblast‐Derived TGFβ1 Protects From Haemorrhage During Wound Healing

**FIGURE 4:**
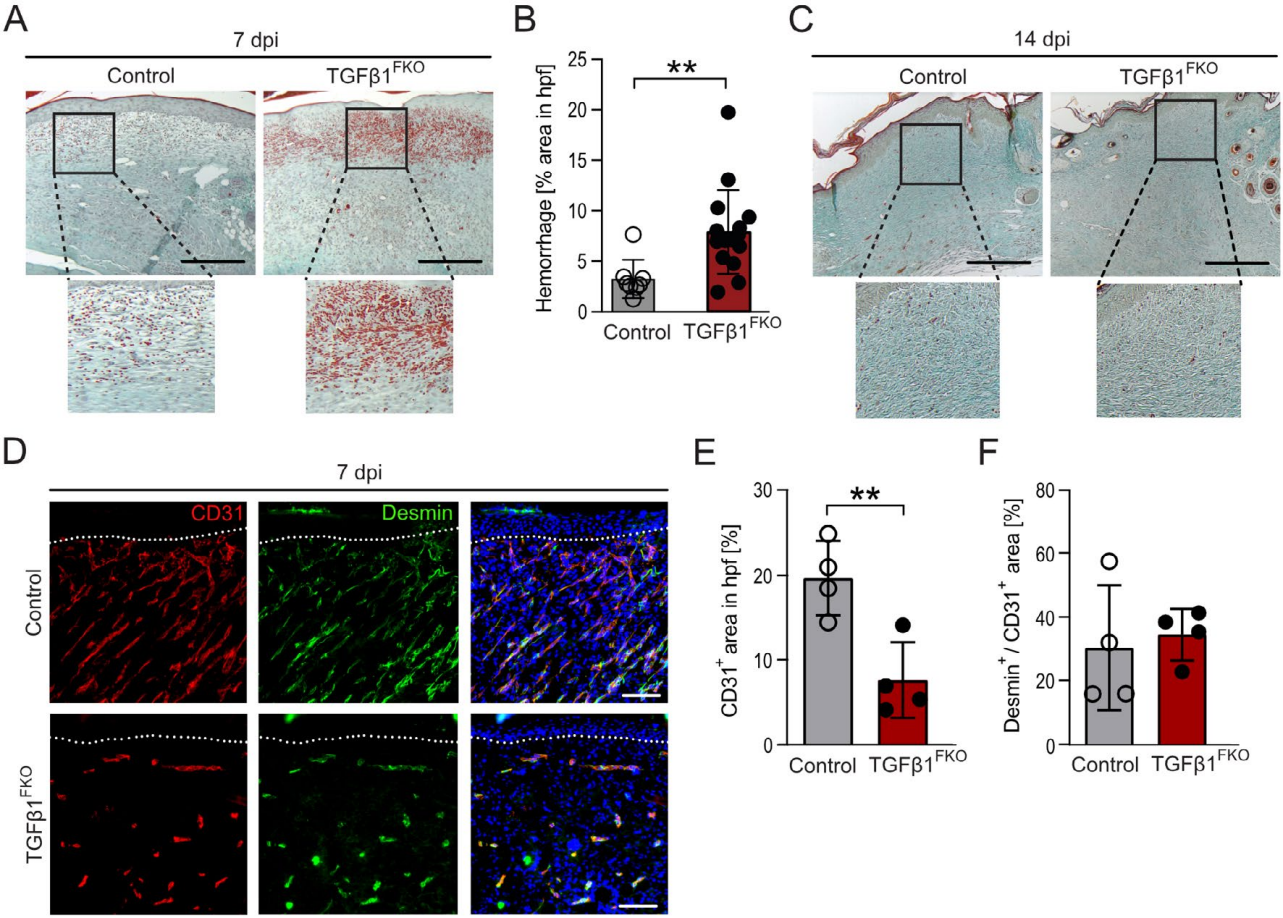
Fibroblast‐derived TGFβ1 protects from haemorrhage during wound healing. Representative Masson's trichrome staining at 7 (A) and (C) 14 dpi in tamoxifen‐treated TGFβ1^FKO^ and control mice and (B) quantification of haemorrhage in wound tissue at 7 dpi. Scale bars = 300 μm. *n* = 8–16 wounds. Data are shown as mean +/− SD. **p < 0.01 by Mann–Whitney U test. (D) CD31 and Desmin immunostainings in wound tissue at 7 dpi of tamoxifen‐treated TGFβ1^FKO^ and control mice. DAPI was used to stain the nuclei. Dotted line underlines the hyperproliferative epithelium. Scale bar: 100 μm. Quantification of (E) the CD31^+^ area per high power field (hpf) and (F) the Desmin^+^ area per CD31^+^ area in wound tissue at 7 dpi of tamoxifen‐treated TGFβ1^FKO^ and control mice. *n* = 4 biological replicates. Data are shown as mean +/− SD. **p < 0.01 by Student's unpaired two‐tailed t test. dpi, days post injury.

To further understand the impact of fibroblast‐derived TGFβ1 on the quality of the granulation tissue, we conducted Masson's trichrome staining on wound sections from TGFβ1^FKO^ and control mice. As already seen in the H&E staining, granulation tissue in wounds from TGFβ1^FKO^ mice was highly hemorrhagic after 7 dpi (assessed by measuring the area covered by erythrocytes), indicating deficient granulation tissue formation and failure of the repair response (Figure 4A,B). Haemorrhages resolved during the time course of repair (Figure 4C). Haemorrhages in wounds of TGFβ1^FKO^ mice resembled the hemorrhagic phenotype we described previously in wound tissue of mice with myeloid cell‐restricted deficiency of IL‐4Rα [43]. The hemorrhagic phenotype in IL‐4Rα mutant mice was accompanied by impaired association of CD31^+^ cells with Desmin^+^ perivascular cells and perturbed vascular morphology [43]. To analyse vascular morphology and coverage of blood vessels with Desmin^+^ perivascular cells, we performed a CD31/Desmin co‐immunostaining on wound sections from TGFβ1^FKO^ and control mice. Analysis in hemorrhagic regions revealed a significantly lower blood vessel density in the granulation tissue of TGFβ1^FKO^ (Figure 4D,E). Moreover, blood vessels in granulation tissue of TGFβ1^FKO^ mice failed to form a branched network that was prominent in granulation tissue of control mice (Figure 4D). The coverage of blood vessels with Desmin^+^ perivascular cells was similar in TGFβ1^FKO^ and control wounds at 7 dpi (Figure 4D,F). At 14 dpi, the blood vessel density was lower in the wounds of both control and TGFβ1^FKO^ mice compared with 7 dpi, with no significant differences between the two genotypes (Figure S1A,C). To prove that the observed vascular phenotype in TGFβ1^FKO^ mice is linked with deficient TGFβ1 signalling, we performed an immunostaining against phospho‐SMAD2 (pSMAD2), which is part of the canonical TGFβ signalling pathway. Indeed, analysis revealed a significantly lower number of pSMAD2^+^ cells in the hemorrhagic regions of TGFβ1^FKO^ mice when compared with the granulation tissue of control mice at 7 dpi (Figure S1D,E). Towards 14 dpi, the number of pSMAD2^+^ cells decreased in the wound tissue of both control and TGFβ1^FKO^ mice, with no significant difference between the two genotypes. Taken together, our findings reveal that the hemorrhagic phenotype in wound tissue of TGFβ1^FKO^ is accompanied by transiently lower blood vessel density and impaired branching of blood vessels.

### 3.5 TGFβ1 Deficiency in Fibroblasts Impairs Gene Expression of Pro‐Angiogenic Factors

**FIGURE 5:**
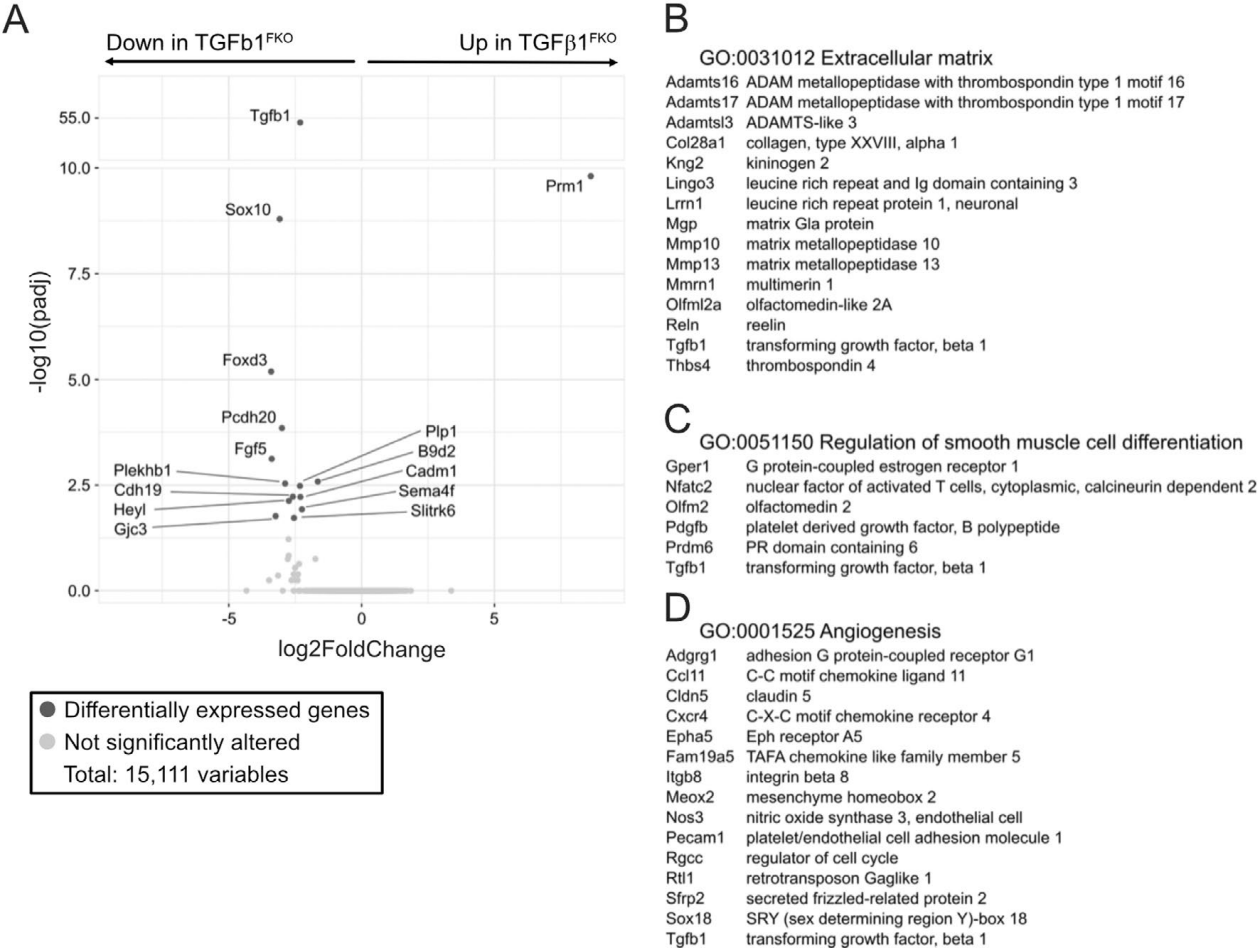
RNAseq analysis in skin fibroblasts isolated from tamoxifen‐treated TGFβ1^FKO^ and control mice. (A) Volcano plot showing all identified genes with log2 fold change of skin fibroblasts isolated from tamoxifen‐treated TGFβ1^FKO^ and control mice against adjusted *p*‐value. Differentially expressed genes (DEG; |fold change| > 1.5 and adjusted *p*‐value < 0.05) in fibroblasts isolated from TGFβ1^FKO^ mice versus controls are shown in green. (B–D) Gene set enrichment analysis showing significantly affected gene ontology terms and the top 15 corresponding genes with lowest log2 fold change.

TGFβ1 itself is known to regulate migration, proliferation, and survival of endothelial cells [44, 45, 46]. We asked whether a lack of TGFβ1 in fibroblasts may lead to impaired expression of other genes critically involved in the regulation of endothelial cell function. We therefore subjected skin fibroblasts isolated from homeostatic skin of adult tamoxifen‐treated TGFβ1^FKO^ and control mice to RNAseq analysis. Analysis of transcriptomes revealed 15 differentially expressed genes (DEG; |fold change| > 1.5 and adjusted *p*‐value < 0.05) in TGFβ1‐deficient fibroblasts versus controls (Figure 5A).

As expected, TGFβ1 expression was absent in fibroblasts isolated from TGFβ1^FKO^ mice. The volcano plot also demonstrated several additional downregulated genes which could be due to the missing autocrine activity of TGFβ1, including FGF5 (Figure 5A). Interestingly, FGF5 has been shown to be a crucial factor for angiogenesis in a 3D microfluidic angiogenesis system [47]. Treatment of human aortic endothelial cells with FGF5 siRNA resulted in significantly reduced vascular sprouting in the microfluidic chamber [47]. Furthermore, GO‐enrichment analysis (Figure 5B–D) showed that several downregulated genes are related to the extracellular matrix, regulation of smooth muscle cell differentiation, and angiogenesis. Besides FGF‐5 and PDGFB, Reln coding for Reelin has been shown to regulate endothelial tube formation [48]. Taken together, we hypothesise that TGFβ1 deficiency impairs vascular sprouting and stability in TGFβ1^FKO^ mice, leading to hemorrhagic granulation tissue.

## 4 Discussion

Fibrosis and scarring following tissue injury are critical processes guaranteeing the survival of all organisms. The underlying processes are regulated by a complex network of different tissue resident cells as well as other cellular players and their precursors that are recruited from the circulation with partially overlapping and redundant activities [1, 3, 4, 49].

TGFβ is a major fibrogenic cytokine [8]. It has various activities depending on the responsive cell type, and complete TGFβ1 knockout mice develop systemic autoimmunity [50]. Regulating TGFβ activity by various mechanisms has been used as a therapeutic approach to modulate fibrotic reactions [51].

Besides fibroblasts, a number of different cell types are potentially capable of releasing TGFβ following tissue injury [52]. Especially in fibrotic reactions, profibrotic macrophages have been associated with activated fibroblasts, and a causal role has been proposed [53]. Furthermore, the cell‐mediated activation of latent TGFβ from the ECM in the fibroblast environment was found to be enhanced in fibrotic diseases [9, 54].

The data provided in this study identify fibroblasts as important producers of TGFβ1 and indicate that fibroblast‐derived TGFβ1 plays a crucial role in bleomycin‐induced fibrosis and wound healing. Although several other mouse models exist that recapitulate certain aspects of human fibrotic reactions [55], the bleomycin model [25] is well suited to analyse the specific role of defined cell types in the process as it leads to an inflammatory fibrotic reaction with multiple cellular players. The restriction of gene inactivation to fibroblasts and related collagen‐producing mesenchymal cells, as well as the temporal control achieved through tamoxifen induction, avoided the generalised autoimmune disease described previously for mice with a complete *Tgfb1* gene ablation [50]. The Cre line used here to target inactivation of *Tgfb1* to fibroblasts is the one published by Christopher P. Denton and coworkers [22], in which Cre recombinase is driven by a minimal promoter of the murine *Col1a2* gene, and Cre activity is dependent on Tamoxifen binding (ER(T)). Activity in fibroblasts is conferred by a far upstream enhancer that was proven to be active in and drive expression of reporter genes [23]. This particular Cre line has been used extensively to drive ablation or overexpression of genes in fibroblasts in vivo [12, 20, 56, 57, 58]. Absence of *Tgfb1* transcripts and TGFβ1 protein in fibroblasts isolated from these mice clearly demonstrated successful ablation of TGFβ1, and our results of reduced fibrosis in the bleomycin model agree well with a report demonstrating induced fibrosis in a mouse in which TGFβ1 signalling was activated in fibroblasts using the same Cre line [59]. However, although the inactivation of *Tgfb1* is controlled by the *Col1a2* promoter fused to the enhancer that restricts Cre activity to fibroblasts, we cannot completely rule out the possibility of other cell types, including fibroblast precursors, being affected. It has recently also been shown that several fibroblast subsets exist, which may be characterised by varying activity of collagen synthesis and therefore can be affected differently [60]. Nevertheless, efficient inactivation of *Tgfb1* in fibroblasts is demonstrated by reduced expression of TGFβ‐dependent downstream target genes throughout the experimental procedure.

Subjecting TGFβ1^FKO^ mice to bleomycin injection clearly demonstrated a reduction of αSMA positive myofibroblasts compared with controls, which is in good agreement with the reduced dermal thickness documented by direct measurement and also by visual evaluation. As no major alteration of the recruitment of inflammatory cellular infiltrate has been noted, we assume that fibroblast derived TGFβ1 is mainly active in a cell autonomous mode. Of note, there were no differences in the density and branching of the blood vessels between fibrotic lesions of control and TGFβ1^FKO^ mice and no signs of haemorrhages. This indicates that the fibroblast released TGFβ1 has a unique activity and cannot easily be compensated by other cellular sources. This might be due to an autocrine activity that has been reported in vitro [61]. This autocrine activity is required for the fibroblast‐to‐myofibroblast conversion in the first days of the development of the fibrotic reaction in vivo. We cannot exclude that this observation is only restricted to the early phases and that other regulatory mechanisms might take over and be more important in later stages.

We then asked the question of whether lack of fibroblast‐derived TGFβ1 might also be critical in other repair situations and used full‐thickness wounds. In this model, we took advantage of a reporter mouse that we generated and which allows us to isolate CD11b^−^CD31^−^eYFP^+^ cells during different phases of tissue repair. These cells represent fibroblasts, and we could demonstrate the presence of fibroblast‐derived TGFβ1 already at 4 days following injury. Although we could also show that *Tgfb1* expression in CD11b^−^CD31^−^eYFP^+^ cells is associated with the expression of several other profibrotic genes, we cannot fully exclude that other wound cells (e.g., epidermal cells) were captured. Given the temporal expression pattern seen in this analysis, we concentrated on analysing wounds at day 7 and day 14, marking a mid and a late phase of the repair response.

At day 7, repair of the injured tissue in the TGFβ1^FKO^ mice clearly differed from controls as documented by reduced wound closure. This is likely due to the reduction in αSMA positive myofibroblasts, which results in a reduced ability of wound contraction. Accordingly, we also observed a larger area of a loose granulation tissue that was characterised by an altered ECM with a reduction of mature collagen fibres, which might represent an altered deposition of collagen I and III. This assumption is based on the use of Picrosirius red staining, in which red collagen fibres reflect mature fibres probably containing mainly collagen [62].

Interestingly, the visually looser granulation tissue formed at day 7 after injury in the TGFβ1^FKO^ mice also displayed excessive haemorrhage; in addition, a disturbed formation of new vessels resulting in a low blood vessel density was detected by Masson's trichrome staining. Impaired branching of blood vessels contributes to the observed phenotype. On one hand, this could be explained by the disturbed macromolecular organisation of the loose connective tissue in the wounds of the mutant mice, which does not allow sufficient mechanical strength to form functional blood vessels. Mechanical forces are thought to be required for neoangiogenesis or sprouting [63]. On the other hand, the conspicuous haemorrhage due to insufficient blood vessel formation and stability could be due to the lack of paracrine activity of TGFβ1 released from fibroblasts. It has been demonstrated that TGFβ signalling is involved in the regulation of differentiation and proliferation of endothelial cells and pericytes [44, 45]. Alternatively, a defective cytokine‐mediated interaction of the fibroblasts with endothelial cells or pericytes could be caused by the lost activity of other growth factors that are under the control of TGFβ1 in fibroblasts. To further investigate this notion, a comparative transcriptome analysis was initiated of dermal fibroblasts isolated from control and TGFβ1^FKO^ mice following tamoxifen treatment. This revealed predominantly downregulation of genes in TGFβ1‐deficient fibroblasts.

Unexpectedly, only a few ECM structural genes were affected, but a clear signature of growth factors was seen. These included FGF5, PDGFβ, and Reelin, which have been associated with the formation of functional blood vessels [48, 64, 65]. Especially, FGF5 has been demonstrated to be a crucial player in regulating angiogenesis [47, 64]. The lack of those factors offers an alternative explanation for the observed vascular abnormalities in the granulation tissue of TGFβ1^FKO^ mice.

These data clearly demonstrated a crucial role of fibroblast derived TGFβ1 in the early steps of wound healing. The observed alterations were transient and the mice completely recovered after 14 days. Probably this can be explained by the temporally controlled interplay of different cellular partners during tissue repair and by the involvement of many other cell types, for example, keratinocytes or immunoregulatory cells that release TGFβ1 in later stages of repair. Further studies using more complex co‐culture systems could add more mechanistic insights. We also cannot exclude the possibility that fibroblasts that have not undergone recombination contribute to the repair process, leading to recovery at 14 dpi.

Our data here demonstrate the role of fibroblasts as a specific cellular source of TGFβ1 with paracrine and/or autocrine activity in two complex situations characterised by disturbed tissue homeostasis in skin. The long‐term consequences of reduced TGFβ1 activity, however, remain unclear and might imply less scarring at the expense of poor angiogenesis. Therefore, the balance between the different activities of TGFβ1 has to be taken into consideration. Nevertheless, interfering with fibroblast‐specific release of TGFβ1 might represent a novel approach to modulate fibrosis that avoids the severe side effects affecting the whole organism. Topical treatment might offer an additional advantage and could be beneficial for the therapy of diseases associated with skin fibrosis.

## Author Contributions

Conceptualization: S.W., K.S., B.E., S.A.E., T.K.; Formal Analysis: S.W., K.S., M.S., A.B., T.Y., I.Z.; Funding Acquisition: B.E., S.A.E., T.K.; Investigation: S.W., K.S., M.S., A.B., T.Y., I.Z.; Methodology: S.W., K.S., M.S.; Project Administration: S.W., B.E., S.A.E., T.K.; Supervision: B.E., S.A.E., T.K.; Validation: S.W., K.S., M.S., B.E., S.A.E., T.K.; Visualisation: S.W., K.S., B.E., S.A.E., T.K.; Writing – Original Draft Preparation: S.W., K.S., M.S., B.E., S.A.E., T.K.; Writing – Review and Editing: all authors.

## Conflicts of Interest

The authors declare no conflicts of interest.

## Supporting information


**Figure S1.** Analysis of CD31, αSMA, and pSMAD2 in wounds of TGFβ1^FKO^ and control mice. (A) Representative αSMA and CD31 immunofluorescence images in wound tissue at 14 dpi from tamoxifen‐treated TGFβ1^FKO^ and control mice. DAPI was used to stain the nuclei. Scale bar = 100 μm. Dotted line underlines the hyperproliferative epithelium. (B) Quantification of αSMA immunofluorescence intensity in wound tissue at 14 dpi from tamoxifen‐treated TGFβ1^FKO^ and control mice. The data are normalised to the control group. *n* = 4 biological replicates. (C) Quantification of the CD31^+^ area per high power field (hpf) in wound tissue at 14 dpi of tamoxifen‐treated TGFβ1^FKO^ and control mice. *n* = 4 biological replicates. (D) Representative pSMAD2 immunostaining in wound tissue at 7 and 14 dpi from tamoxifen‐treated TGFβ1^FKO^ and control mice. Scale bar = 100 μm. Dotted line underlines the hyperproliferative epithelium. (E) Quantification of pSMAD2^+^ cells in the granulation tissue of TGFβ1^FKO^ and control mice at indicated timepoints. *n* = 4–5 biological replicates. Data are shown as mean ± SD. **p.

## Data Availability

The data that supports the findings of this study are available in the supplementary material of this article.
