# Supplementary figures and images for: Fibroblast‐Derived TGFβ1 Regulates Skin Repair and Fibrosis
Source: Wound Repair Regen. 2025 Jul 13;33(4):e70065. doi: 10.1111/wrr.70065 (PMC12256102; doi:10.1111/wrr.70065)

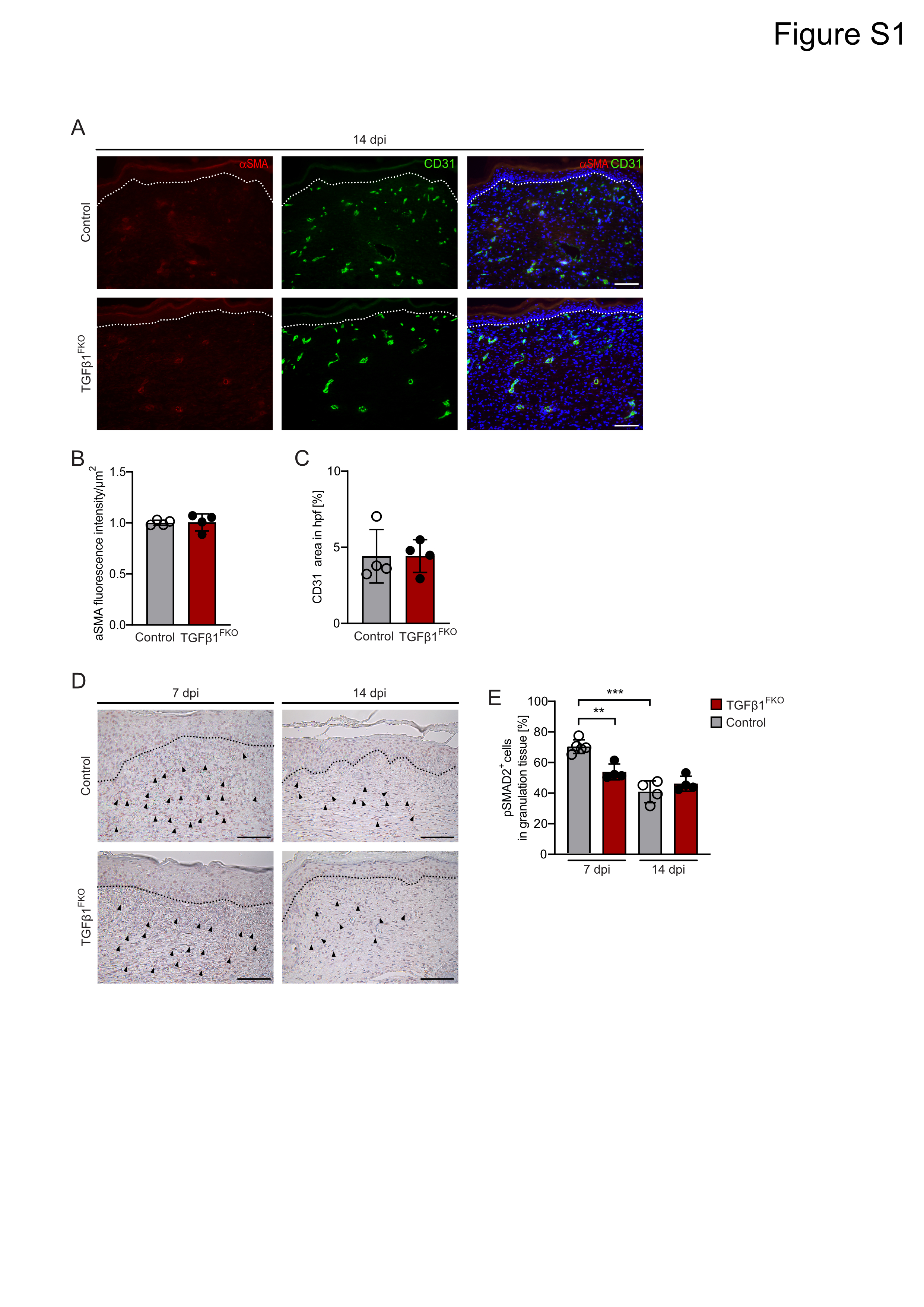

Supplement: Supplementary file 1 — Figure S1. Analysis of CD31, αSMA, and pSMAD2 in wounds of TGFβ1FKO and control mice. (A) Representative αSMA and CD31 immunofluorescence images in wound tissue at 14 dpi from tamoxifen‐treated TGFβ1FKO and control mice. DAPI was used to stain the nuclei. Scale bar = 100 μm. Dotted line underlines the hyperproliferative epithelium. (B) Quantification of αSMA immunofluorescence intensity in wound tissue at 14 dpi from tamoxifen‐treated TGFβ1FKO and control mice. The data are normalised to the control group. n = 4 biological replicates. (C) Quantification of the CD31+ area per high power field (hpf) in wound tissue at 14 dpi of tamoxifen‐treated TGFβ1FKO and control mice. n = 4 biological replicates. (D) Representative pSMAD2 immunostaining in wound tissue at 7 and 14 dpi from tamoxifen‐treated TGFβ1FKO and control mice. Scale bar = 100 μm. Dotted line underlines the hyperproliferative epithelium. (E) Quantification of pSMAD2+ cells in the granulation tissue of TGFβ1FKO and control mice at indicated timepoints. n = 4–5 biological replicates. Data are shown as mean ± SD. **p. [file WRR-33-0-s001.jpg]
